# Metaphenotypes associated with recurrent genomic lineages of *Campylobacter jejuni* responsible for human infections in Luxembourg

**DOI:** 10.3389/fmicb.2022.901192

**Published:** 2022-09-07

**Authors:** Morgane Nennig, Arnaud Clément, Emmanuelle Longueval, Thierry Bernardi, Catherine Ragimbeau, Odile Tresse

**Affiliations:** ^1^Epidemiology and Microbial Genomics, Laboratoire National de Santé, Dudelange, Luxembourg; ^2^UMR-1280 PhAN, INRAE, Nantes, France; ^3^BioFilm Control, Biopôle Clermont-Limagne, Saint-Beauzire, France

**Keywords:** *Campylobacter jejuni*, aerobic acclimation, adhesion capacity, biofilm formation, oxidative stresses, recurring lineages, metaphenotype

## Abstract

*Campylobacter jejuni* is a leading cause of foodborne illnesses worldwide. Although considered fragile, this microaerophilic bacterium is able to survive in various challenging environments, which subsequently constitutes multiple sources of transmission for human infection. To test the assumption of acquiring specific features for adaptation and survival, we established a workflow of phenotypic tests related to the survival and the persistence of recurrent and sporadic strains. A representative collection of 83 strains isolated over 13 years from human, mammal, poultry, and environmental sources in Luxembourg, representing different spreading patterns (endemic, epidemic, and sporadic), was screened for survival to oxidative stresses, for acclimating to aerobic conditions (AC), and for persistence on abiotic surfaces. Using the cgMLST Oxford typing scheme for WGS data, the collection was classified into genomic lineages corresponding to host-generalist strains (lineages A and D, CC ST-21), host-specific strains (lineage B, CC ST-257 and lineage C, CC ST-464) and sporadic strains. We established that when a strain survives concentrations beyond 0.25 mM superoxide stress, it is six times more likely to survive hyperoxide stress and that a highly adherent strain is 14 times more likely to develop a biofilm. Surprisingly, more than half of the strains could acclimate to AC but this capacity does not explain the difference between recurrent genomic lineages and sporadic strains and the survival to oxidative stresses, while recurrent strains have a significantly higher adhesion/biofilm formation capacity than sporadic ones. From this work, the genomic lineages with more stable genomes could be characterized by a specific combination of phenotypes, called metaphenotypes. From the functional genomic analyses, the presence of a potentially functional T6SS in the strains of lineage D might explain the propensity of these strains to be strong biofilm producers. Our findings support the hypothesis that phenotypical abilities contribute to the spatio-temporal adaptation and survival of stable genomic lineages. It suggests a selection of better-adapted and persistent strains in challenging stress environments, which could explain the prevalence of these lineages in human infections.

## Introduction

*Campylobacter* is the leading cause of bacterial gastroenteritis worldwide (World Health Organization [WHO], 2013). The related disease, campylobacteriosis, has been the most frequently reported zoonosis in the European Union (EU) since 2005 (European Food Safety Authority [EFSA], and European Centre for Disease Prevention and Control [ECDC], 2021). Its incidence has increased throughout Europe, with a stabilization phase during 2015–2019 (59.7 cases per 100,000 inhabitants in 2019). In Luxembourg, an upward trend was also observed from 42.6 cases per 100,000 inhabitants recorded in 2005 to a peak of 158.8 cases per 100,000 inhabitants in 2014, associated with the largest outbreak ever recorded on the national scale. The prevalence has remained relatively high with an incidence of 103.8 per 100,000 inhabitants in 2017 and 2018, making this country a representative of a full surveillance system (European Food Safety Authority [EFSA], and European Centre for Disease Prevention and Control [ECDC], 2006, 2015, 2019a,b; Nennig et al., 2021).

More than 80% of reported human cases are related to *Campylobacter jejuni*. *C. jejuni* is asymptomatically carried in the digestive tract of animals, mainly wild and domestic birds (Mughini-Gras et al., 2012; European Food Safety Authority [EFSA], and European Centre for Disease Prevention and Control [ECDC], 2021). Campylobacteriosis is often associated with the consumption of contaminated food such as undercooked poultry, unpasteurized milk, and cross-contaminated food (e.g., vegetables) (Boysen et al., 2014; MacDonald et al., 2015). Some studies have reported cases of campylobacteriosis due to direct contact with environmental sources such as pets, recreational water (e.g., swimming), or professional exposure (e.g., humans in poultry farms and slaughterhouses) (Mughini-Gras et al., 2013; Vegosen et al., 2015; Ravel et al., 2016; Montgomery et al., 2018; Duijster et al., 2019). These reports suggest the presence of other reservoirs involved in the transmission routes of *C. jejuni*.

From a One Health perspective, integrated surveillance of *C. jejuni* genetic profiles has been implemented in Luxembourg since 2005. In a previous study, we have performed genomic comparison analyses using different gene-by-gene typing schemes designed for *C. jejuni*. Our findings revealed that some genotypes, regularly isolated from patients in Luxembourg, could be segregated into distinct genomic lineages displaying either endemic, epidemic, or diffused outbreak patterns in their temporal distribution. In addition, their persistence was characterized by a more stable genome over the years (Nennig et al., 2021). As the transmission from primary reservoirs to the human requires adaptation capabilities to overcome harmful, even lethal conditions, this study aimed at investigating phenotypical features that could favor survival, adaptation, and persistence. As a microaerophilic and capnophilic microorganism, *C. jejuni* requires an atmosphere reduced in oxygen (O_2_; ∼5%) and enriched in carbon dioxide (CO_2_; ∼10%) for growth, and it cannot survive under atmospheric oxygen levels for more than a few hours (Garénaux et al., 2007; Macé et al., 2015). *C. jejuni* has to develop mechanisms to ensure its survival under stressful conditions in order to persist in the food environment (i.e., on food, food plants, and carcasses), throughout the food chain, and to trigger an infection in humans (Bronowski et al., 2014; Yahara et al., 2017). Considering its fastidious nature for growth, biological advantages that may be involved in its survival are limited. For instance, the absence of RpoS factor could limit responses to general stress (Parkhill et al., 2000). Carbon source assimilation is also a limiting factor in the adaptation capabilities of *C. jejuni*. Although a genomic island was identified in some strains to challenge enteric bacteria by uptaking and utilizing L-fucose (Stahl et al., 2012) and 1.7% of over 6,000 *C. jejuni* strains analyzed were able to restore the glycolysis *via* the Entner–Doudoroff pathway (Vegge et al., 2016), most of the strains remain asaccharolytic, which reduces niches for multiplication. Then, amino acids constitute the main carbon source for *C. jejuni*. Some adaptations to hyper- or low-osmotic environments have been described for *C. jejuni*; however, they are temperature-dependent and remain limited (Burgess et al., 2016). According to Pascoe et al. (2015) and Karki et al. (2018), the primary strategies used by *Campylobacter* for its persistence are the formation of biofilms and the ability to survive oxidative stresses (Pascoe et al., 2015; Karki et al., 2018). These adaptive capabilities were described for only a few strains and were mainly assessed on well-studied strains (Turonova et al., 2015; Oh et al., 2016; Park et al., 2021).

In broad terms, biofilms are defined as multicellular layers of bacteria, enshrouded in a matrix of extracellular polymeric substances (EPS) often attached to surfaces (Donlan and Costerton, 2002). Biofilms formed by *C. jejuni* could be generated by cells attached to inert surfaces, self/mixed-aggregates floating within a liquid culture, or a pellicle layer at the liquid-gas interface (Joshua, 2006). Biofilm development requires five successive coordinated steps: (i) the biofilm initiation that is composed of the reversible attachment of bacterial cells to a surface (adherence) followed by the irreversible attachment (adhesion), (ii) the biofilm growth involving the formation of micro-colonies, (iii) the maturation of the biofilm, and (iv) the detachment and dispersion of the cells (Annous et al., 2009). Interestingly, the development of *C. jejuni* biofilms is enhanced under aerobiosis rather than under optimal microaerobic gas concentrations (Reuter et al., 2010; Sulaeman et al., 2012; Turonova et al., 2015). *C. jejuni* can form monospecies biofilms or colonize pre-established multispecies biofilms (Hanning et al., 2008; Ica et al., 2012; Teh et al., 2019). Additionally, bacteria enclosed in biofilms are a thousand times more resistant to antimicrobial agents than planktonic counterparts (Fux et al., 2005). *Campylobacter* biofilms on food processing surfaces in production lines protect the pathogen from cleaning and sanitizing measures, resulting in further contamination of food products, which contributes to its spread (Nguyen et al., 2012; Brown et al., 2014). Therefore, the bacterial biofilm is recognized as a key player in the survival of *C. jejuni* in various ecological niches.

Oxidative stress results from the formation of highly reactive oxygen species (ROS) due to the incomplete reduction of oxygen. The ROS includes the superoxide anion (O_2_^–^), hydrogen peroxide (H_2_O_2_), and hydroxyl radical (HO^.^) (Imlay, 2008). They degrade and alter protein functions and cause irreversible damage to lipids and DNA (Fisher and Stadtman, 1992; Yamasaki et al., 2004). *C. jejuni* is exposed to highly variable dioxygen concentrations during its life cycle: throughout the food chain as well as in the host guts as the immune system produces H_2_O_2_ to kill microbes (Melo et al., 2019). Although *C. jejuni* lacks specific regulators, such as SoxRS or RpoS that are present in most Gram-negative bacteria, it encodes oxidative defense enzymes including the superoxide dismutase SodB, the major subunit of the alkyl hydroperoxide reductase AhpC, and the catalase KatA (Grant and Park, 1995; Garénaux et al., 2007; Atack et al., 2008). *C. jejuni* also encodes other antioxidant enzymes, such as the thiol peroxidase Tpx and the bacterioferritin co-migratory protein Bcp: both playing a role in the protection of *C. jejuni* against oxidative stresses but could also contribute to aerotolerance (Atack et al., 2008). Indeed, aerotolerance is closely related to the defense against oxidative stresses, as aerobic exposure results in ROS accumulation (Oh et al., 2015b). Some strains of *C. jejuni* have been reported to have different tolerance levels in the presence of atmospheric oxygen concentrations (Kaakoush et al., 2007; Rodrigues et al., 2015, 2016; Oh et al., 2017). These aerotolerant strains are frequently prevalent in chickens, and most of them belong to genotypes that are often implicated in human infection and outbreaks (Oh et al., 2015a; Lee et al., 2019). Conversely, limited studies investigated the acclimation of *Campylobacter* to aerobic conditions (AC) (Rodrigues et al., 2015; O’Kane and Connerton, 2017).

This study aimed to investigate the phenotype of 83 strains of *C. jejuni*, isolated from multi-hosts between 2005 and 2018 in Luxembourg, including representative strains of the stable genomic lineages previously described. The objective was to determine whether these strains could (i) survive oxidative stresses, (ii) adapt to AC, and (iii) persist on inert surfaces. These specific traits were investigated as they could be potentially linked to spatio-temporal strain survival and persistence. Correlations to epidemiological profiles were statistically investigated and when a correlation was found, a functional genomic analysis was performed based on the Venn diagram resulting from high resolutive wgMLST INNUENDO comparisons.

## Materials and methods

### Bacterial strains and growth conditions

In this study, *C. jejuni* strains were selected based on the frequency of their genotypes (defined by extended MLST and/or cgMLST) in human infections. From our genomic surveillance system, we identified diffused outbreaks linked to the persistence over time of some genetic profiles (Nennig et al., 2021). These genomic lineages were not only associated with human gastroenteritis cases but also with mammals, poultry, and the environment. Therefore, the strain collection was built up to reflect the circulating strains in Luxembourg. Fifty-two strains belonging to the four genomic lineages with a more stable genome (Nennig et al., 2021) were selected for their recurrence in human infections between 2006 and 2018 (i.e., displaying either endemic, epidemic/micro-epidemic, and emergent patterns). As an outgroup, 19 sporadic human clinical strains with previously characterized WGS data (Nennig et al., 2021) were added. The sporadic profile was defined when a unique ST-gyrA-porA profile occurred only once between 2011 and 2018. In addition, 10 sporadic strains isolated from environmental sources were included. The environmental source was defined when the isolate had never been detected in human infections in Luxembourg. The strain NCTC 11168 was also included as a well-studied reference strain and the atypical aerotolerant strain Bf as a control (Rodrigues et al., 2015). Altogether 83 strains were investigated in this study (Supplementary Data 1).

All of the strains were stored at −80°C in FBP medium (a combination of ferrous sulfate, sodium metabisulfite, sodium pyruvate, and glycerol) as described by Gorman and Adley (2004). Before each experiment, a loopful of a frozen culture of each strain was spread on BD™ *Campylobacter* Bloodfree Selective Medium (Becton Dickinson GmbH, Deutschland) and incubated under microaerobic conditions (MAC), namely MAC (6% O_2_, 3.6% CO_2_, 3.6% H_2_, and 86.8% N_2_) at 42°C for 48 h. MAC was obtained using an Anoxomat Mark II jar-gas distributing system (Mart^®^ Microbiology BV, Netherlands). Then, a subculture was obtained from one colony on the same medium and incubated for 16 h in the conditions mentioned earlier.

### Genotyping data of *C. jejuni* isolates and phylogenetic analyses

The environmental strains were processed by WGS and were analyzed using the cgMLST Oxford scheme composed of 1,343 loci as described previously (Cody et al., 2017; Jolley et al., 2018; Nennig et al., 2021). This typing scheme was implemented in SeqSphere + v6.1 (Ridom GmbH, Münster, Germany) to compare strains using the same previously defined in-house nomenclature, i.e., with a cut-off alert set at 11. Briefly, the paired-end raw reads were *de novo* assembled using Velvet Optimizer v1.1.04 and quality control (QC) criteria included a maximum number of 150 contigs (average = 62), assemblies with >30× coverage (average = 81), and no more than 5% of missing targets in cgMLST (average = 98% of good targets found). Few exceptions of QC outside the range were observed from environmental strains with more exotic genetic profiles (two strains with a percentage of targets found ≥88.5%). All QC data from WGS are available in Supplementary Data 1. MLST data were extracted from cgMLST data to determine the STs and CCs. Supplementary raw reads have been uploaded to ENA and are available under the accession project number PRJEB51093. The graphical representation of the phylogenetic analyses was constructed through a UPGMA tree based on the cgMLST allelic profiles generated by the typing scheme described previously. The “pairwise ignoring missing values” was used as the setting. For wgMLST INNUENDO, assemblies were performed using the INNUca pipeline v.4.2.1 (Machado et al., 2017; Silva et al., 2018) and only profiles with no more than 2% of missing loci in cgMLST INNUENDO was applied as pre-filter QC for the identification of unique targets from the pan genome (Nennig et al., 2021) and paragraph 2.7.

### Survival of *C. jejuni* isolates in oxidative stress conditions

The strains were exposed to three concentrations (0.12, 0.25, and 0.50 mM) of paraquat (PQ) or hydrogen peroxide (H_2_O_2_) solutions to induce superoxide and peroxide stresses as previously described by Rodrigues et al. (2015), with the following modifications: after resuspension, the cultures were standardized to an optical density (OD) at 600 nm (OD_600nm_) of 1.00 (≈10^9^ CFU.mL^–1^) and diluted in sterile peptone water to obtain a bacterial suspension containing approximately 10^6^ CFU.mL^–1^. The initial culture was plated out for concentration determination: an average concentration of 9.5 ± 0.3 log_10_ CFU.mL^–1^ (*n* = 83) was determined. The bacterial suspensions were incubated for 45 min at 42°C in a modular atmospheric controlled system cabinet under MAC. The capacity of the strains to resist oxidative stresses was determined by plating dilutions on Karmali agar plates using the microdroplet technique as described by Rodrigues et al. (2015). The survival rate was calculated as the viable cell concentration obtained after oxidative stress divided by the initial concentration. The experiment was performed in three technical replicates, and an average of the triplicates values was calculated. A control was carried out in parallel with each test, containing the same bacterial inoculum but without the addition of PQ or H_2_O_2_.

### Acclimation to aerobic conditions

Cells were prepared for acclimation to AC by growing them in MAC on a charcoal-based selective medium for 48 h according to Rodrigues et al. (2015). Colonies were then harvested and resuspended in sterile peptone water and standardized to an OD_600nm_ of 1.00 (≈10^9^ CFU.mL^–1^). For each bacterial suspension, 1 mL containing approximately 10^9^ CFU.mL^–1^ was inoculated onto charcoal-based selective agar plates and incubated at 37°C under AC for 48 h. Then, all the recovered colonies were subcultured two times successively on charcoal and blood-free Columbia base agar (Biokar, France) plates in parallel and incubated at 37°C for 48 h in AC. The inoculum, initially resuspended in sterile peptone water, was plated out for concentration determination: the average concentration was 9.5 ± 0.3 log_10_ CFU.mL^–1^ (*n* = 83). When growth was obtained, colonies were submitted to the MALDI-TOF Biotyper Microflex LT (Bruker Daltonics, Germany) for species identification, associated with *in vitro* diagnostic and research use only databases. A minimum matching score of 2 was required for each *C. jejuni* strain, confirming the species. The leftover was stored at −80°C in standard conditions. In addition, the genome of seven strains able to acclimate to AC was sequenced using Illumina technology using the protocol as above mentioned (see section “Genotyping data of *C. jejuni* isolates and phylogenetic analyses”).

### Adhesion to an inert surface and biofilm formation

The adhesion capacity to an inert surface of *C. jejuni* strains was determined using the BioFilm Ring Test^®^ (KITC004, BioFilm Control, France). The protocol was adapted from the one described by Sulaeman et al. (2010). Briefly, each culture was harvested and resuspended in ultrapure water (UPW) (Water UHPLC-MS, code product 15339865, Thermo Scientific); the OD_600 nm_ was adjusted to 1.00 (≈10^9^ CFU.mL^–1^). The initial concentration estimated at 10^9^ CFU.mL^–1^ was determined by plate counting. The toner solution (TON004) containing magnetic beads was mixed for 1 min and added (10 μL.mL^–1^) to each calibrated bacterial suspension. A quantity of 200 μL of bacterial suspension mixed with the beads was distributed in 96-well microtiter plates, with three wells per strain (technical replicates). The plates were incubated for 2 h in AC at 42°C. After incubation, an inert opaque oil was used as a contrast liquid, and a few drops covered the bacterial solution in the wells. Then, microplates were magnetized for 1 min with the magnet block and read using the dedicated Scan Plate Reader operated with a computer through BIOFILM CONTROL ELEMENTS^®^ 3 software in a custom mode (BioFilm Control, France). The calculated numerical value, called Biofilm Formation Index (BFI), measures the aggregation density of the toner beads under the effect of a magnetic field generated by a magnet. As described by Sulaeman et al. (2010), the ΔBFI (BFI_control_ − BFI_sample_) was calculated to express the adhesion capacity of strains (Sulaeman et al., 2010). Three wells were filled with UPW and beads only for each microplate, representing the negative control. For each strain, three biological replicates with three technical replicates each were performed. Values were considered valid when the standard deviation between technical replicates did not exceed 1.5.

The biofilm development capacity was assessed from a new simple and reliable *in vitro* procedure standardized to characterize biofilm-producing *C. jejuni* strains based on the BioFilm Ring Test^®^ technology. A fresh overnight culture of bacteria grown on BD™ *Campylobacter* Bloodfree Selective medium was harvested and resuspended in ultrapure water; the OD_600 nm_ was adjusted to 1.00 (≈10^9^ CFU.mL^–1^). The initial concentration was determined by plate counting. A quantity of 200 μL of bacterial suspension was distributed in 96-well microtiter plates, with three wells per strain (technical replicates). Three wells containing ultrapure water and microbeads mix without microbial cells were used as negative controls for each experiment. Preliminary trials were performed to identify two strains from the collection as standard and quality controls. Strain Bf was used for positive biofilm formation and Camp052 for negative biofilm formation. Preliminary assays were performed to evaluate the biofilm formation dynamic with monitoring at 20, 22, 24, 26, and 30 h for 24 strains, based on Turonova et al. (2016) results. The test was refined, and a reading at 22 h of incubation was selected, as it was the reliable incubation time to distinguish biofilm-producing strains from late-biofilm-producing or no-biofilm-producing strains. The plates were prepared and incubated for 2 h in AC at 42°C. A 190 μL volume of the supernatant was then removed and replaced with a fresh tenfold dilution of Brain Heart Infusion (BHI; BioFilm Control, France) combined with magnetic beads (10 μL.mL^–1^). The plates were then re-incubated for 20 h in AC at 42°C. They were read using the same procedure as the adhesion tests. Each strain was analyzed in technical and biological replicates. Technical replicates were considered valid when the standard deviation between recorded values did not exceed 1.5.

### Fluoroquinolone resistance

Predicted antimicrobial resistance (AMR) patterns were established using the analysis of the *gyrA* alleles as previously described by Ragimbeau et al. (2014) as it is the main gene involved in fluoroquinolone resistance of *C. jejuni*.

### *In silico* identification of the unique targets from the pan-genome

The specific targets belonging to the four different lineages were previously determined using the wgMLST INNUENDO typing scheme (Nennig et al., 2021). As targets may be only a part of a gene, their presence, synteny, and organization were explored using the Platform MicroScope^1^ (Vallenet et al., 2017, 2020). Using the manual annotation of strains uploaded on this platform, protein identification was performed whenever it was possible. This analysis was completed and the biological functions of the detected proteins were inferred using KEGG and UniProt (Kanehisa et al., 2016; The UniProt Consortium, 2021). The presence of the type VI secretion system (T6SS) core components was investigated in the recurrent strains of our collection. As a fully assembled T6SS is composed of a minimal of 13 core components, the list of genes of interest was extracted from Zoued et al. (2014): *tssJ*, *tssK*, *tssL*, *tssM*, *tssA*, *tssB*, *tssC*, *hcp*, *tssE*, *tssF*, *tssG*, *clpV*, and *vgrG*. Nucleotide and amino acid sequences of the genes were collected from the NCBI RefSeq genome database, from the *C. jejuni* 488 strain (Liaw et al., 2019). BlastN and blastP were performed using the Platform MicroScope for the first screening of the strains. Geneious^®^ v.11.1.3^2^ was used to map specific sequences against whole genome of the strains, and BioEdit v.7.0.5.3^3^ was used to align the target sequences to visualize potential mutations.

### Statistical analyses

Statistical analyses included the comparison of groups of strains, according to the survival to oxidative stresses (H_2_O_2_ and PQ), the acclimation to AC, the adhesion capacity, the biofilm formation ability, the antimicrobial susceptibility, the recurrence, and belonging to a particular genomic lineage. The results were analyzed with JMP v.15 software (SAS Institute Inc., Cary, NC, United States), and Microsoft^®^ Excel^®^ 2016 (v.16.0.5239.1001), using the chi-squared test for qualitative variables and relative risk calculation and variance analysis (ANOVA) for quantitative variables and multiple comparisons. The significance level was determined at 95%, *p* < 0.05 considered as significant. When multiple comparisons were performed, the confidence levels for each comparison performed had to be higher to be validated, so that the result of the multiple comparisons meets the 95% confidence level. For this purpose, the Tukey–Kramer method was used to keep the alpha risk at 5%. Distributions were displayed using box and whisker diagrams for graphical representation. The horizontal lines from bottom to top represent the minimum, the first quartile, the median, the third quartile, and the maximum.

## Results

### Genetic diversity within the strain collection

**FIGURE 1:**
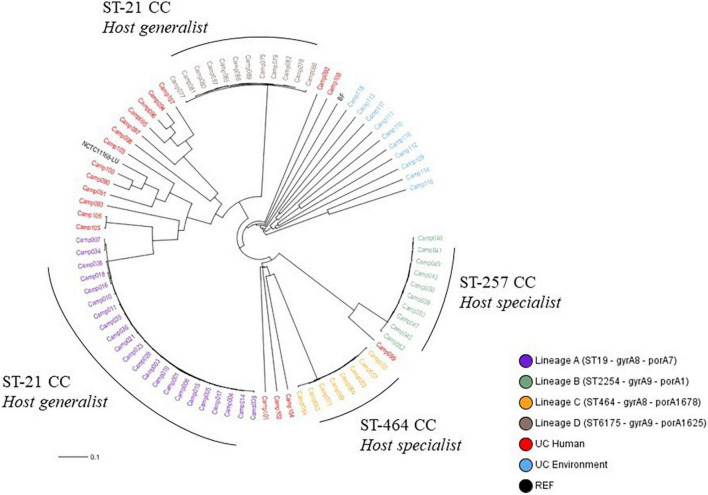
UPGMA phylogenetic tree for 83 *Campylobacter jejuni* isolates based on the cgMLST Oxford allelic profiles (1,343 loci). CC refers to Clonal Complex, NCTC11168 is a reference strain, and Bf was used as a positive control for aerobic acclimation and biofilm formation, ST to sequence type, and UC to unique combinations.

The cgMLST Oxford scheme, previously assessed to classify *C. jejuni* strains with a validated cluster-alert distance (Nennig et al., 2021), was used in this study for the phylogenetic analysis of our strain collection. Overall, 22, 10, 8, and 12 strains belonged to lineages A, B, C, and D, respectively. In addition, 29 strains from clinical and environmental sources displayed a unique genomic profile (Unique Combination, UC) as well as NCTC 11168 and Bf strains (Figure 1). According to the distance matrix (Supplementary Data 2), strains belonging to lineage A had at least 428 allele differences (AD) from the other strains. Strains belonging to lineages B, C, and D were distant from the rest of the collection with at least 305, 1,019, and 912 AD, respectively. These results corroborated that the four lineages were genetically distinct. Furthermore, strains, classified as human UC, presented at least 109 AD with other strains, except Camp015 and Camp106, which are distant from 12 AD only. As the cluster alert implemented for the cgMLST Oxford was at 11 AD (Nennig et al., 2021), these two strains were therefore considered as distinct genetic profiles. The environmental strains with unique combinations had at least 642 AD with the other strains. They were not related to any persistent genomic lineages. The reference strain, NCTC 11168, and the Bf strain were distant from the others at least at 247 and 1,177 AD, respectively. According to Dingle et al. (2001)’s strain classification by CCs, CC ST-21 covers at least the lineages A and D which are considered as host-generalist lineages, while the CC ST-257 corresponds to the lineage B and the CC ST-464 to the lineage C, both designed as host-specialist lineages by Sheppard et al. (2014) (Figure 1).

### Phenotypical characterization of *C. jejuni* strains

#### Survival to oxidative stresses

**FIGURE 2:**
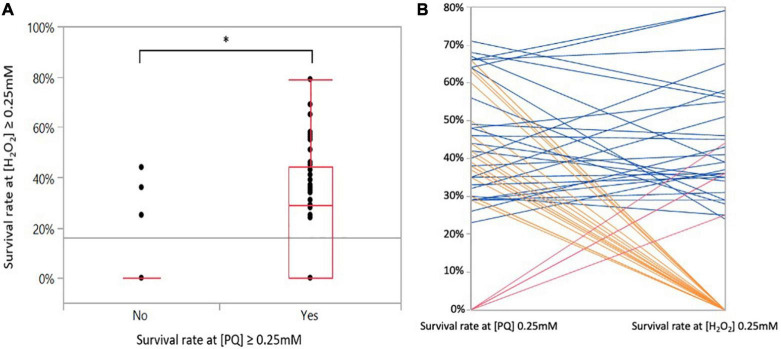
Correlations between superoxide and hyperoxide stress responses. **(A)** Survival rate of the 83 *C. jejuni* strains exposed at 0.25 mM H_2_O_2_ as a function of their survival to 0.25 mM PQ. Statistical significance was determined using the ANOVA test (*p ≤ 0.05). **(B)** Relationship between survival at 0.25 mM PQ and H_2_O_2_. The parallel coordinates indicate strains that show similar survival responses or not to both stresses. Each line corresponds to a strain exposed to PQ (left *y*-axis) and to H_2_O_2_ (right *y*-axis). Blue lines correspond to strains surviving both stresses at a concentration of 0.25 mM (*n* = 27), orange lines to strains surviving at 0.25 mM of PQ but not at 0.25 mM H_2_O_2_ (*n* = 20), and red lines to strains surviving at 0.25 mM H_2_O_2_ but not at 0.25 mM PQ (*n* = 4).

Two pro-oxidant reagents, PQ and H_2_O_2_, were used to induce superoxide and hyperoxide stresses, respectively. Overall, diverse responses were observed among strains after a short contact with increasing concentrations of PQ and H_2_O_2_. A total of 18, 28, and 17 strains survived 0.12, 0.25, and 0.50 mM of PQ, respectively, and there was no survival in 20 out of the 83 strains in the presence of 0.12 mM of PQ (Table 1). A total of 27, 23, and 8 strains survived 0.12, 0.25, and 0.50 mM of H_2_O_2_, respectively. In the presence of 0.12 mM of H_2_O_2_, 25 strains could not survive (Table 1). Altogether, 38 and 52 strains were not able to cope with a concentration equal to or above 0.25 mM of PQ and H_2_O_2_, respectively. In contrast, 45 and 31 strains could deal with higher concentrations (0.25 or 0.50 mM) of PQ and H_2_O_2_, respectively. Consequently, strains were classified as ‘susceptible’ or ‘resistant’ according to their oxidative stress responses (Table 1). PQ is not significantly more harmful than H_2_O_2_. Survival at 0.25 mM PQ was significantly correlated with survival at 0.25 mM H_2_O_2_ (ANOVA, *p* < 0.0001) (Figure 2A) with 27 strains surviving both stresses at 0.25 mM (Figure 2B). Consistent with this correlation and based on the calculation of the relative risk, it was highlighted that when a strain survives a concentration equal to or above 0.25 mM PQ, it is six times more likely to survive H_2_O_2_ at the same concentration (chi-squarest test, *p* < 0.0001).

**TABLE 1:** Classification of 83 *Campylobacter jejuni* isolates according to their response to superoxide and hyperoxide stresses.

Concentrations tested	Number of strains surviving the superoxide stress (PQ)	Number of strains surviving the hyperoxide stress (H_2_O_2_)	Classification
<0.12 mM	20	25	Susceptible
0.12 mM	18	27	
0.25 mM	28	23	Resistant
0.50 mM	17	8	

#### Acclimation to aerobic conditions

The acclimation assay was selected to determine the capacity of multiplication of *C. jejuni* on solid media under AC, based on previous results obtained for the strain *C. jejuni* Bf (Rodrigues et al., 2015). Gradual exposure to AC of a calibrated inoculum, resulting in an increasing number of colonies after three consecutive subcultures, was validated for acclimation capability. The results were expressed as follows: in the absence of colonies after the first subculture, the strains were defined as non-acclimated to AC (NAAC). In the case of growth only on charcoal-based selective medium after three subcultures but not on charcoal-blood free medium, an intermediate group of strains was classified as semi-acclimated to AC (SAAC). When colonies were detected after three subcultures on both media, the strains were classified as acclimated to AC (AAC). Unexpectedly, the results indicated that 10 strains could acclimate to AC, irrespective of the solid medium used, and 33 could acclimate after cultivation on the selected medium. The other 40 strains did not show any acclimation capability. Altogether, more than half of the strains (*n* = 43) were able to grow and multiply under AC, while the others could not (Table 2). When acclimated cells were used after freezing storage, none of them were able to grow again after direct submission to AC. Acclimation for these strains could be recovered only by using the same acclimation protocol with the first passage in MAC. The acclimation capability among the strains could not be associated with the isolation sources (animal, human, or environment) (data not shown). WGS data from 6 out of 10 AC-acclimated strains were analyzed before and after acclimation to AC using the cgMLST Oxford scheme (1,343 targets). The core genome was analyzed as we assumed that the acclimation mechanism is the same for each strain of *C. jejuni* able to develop this capability. According to the generated distance matrix, the four strains Bf, Camp016, Camp018, and Camp036 were identical in alleles before and after acclimation to AC. Only one difference in allelic profile was recorded for Camp022 and Camp098 (Supplementary Data 3). The altered target corresponded to CAMP046 in the cgMLST Oxford, and it is related to the Cj0276 locus, also named *mreB*. The two-point mutations at the nucleotide level are non-synonymous, leading to amino acid transition (G583A → Asp195Asn for Camp022 and A224G → His76Arg for Camp098).

**TABLE 2:** Classification of 83 *C. jejuni* isolates submitted to gradual acclimation to aerobic conditions (AC).

Level of acclimation	Number of strains
Non-acclimated to AC (NAAC)	40
Semi-acclimated to AC (SAAC)	33
Acclimated to AC (AAC)	10

#### Adhesion and biofilm formation ability

**FIGURE 3:**
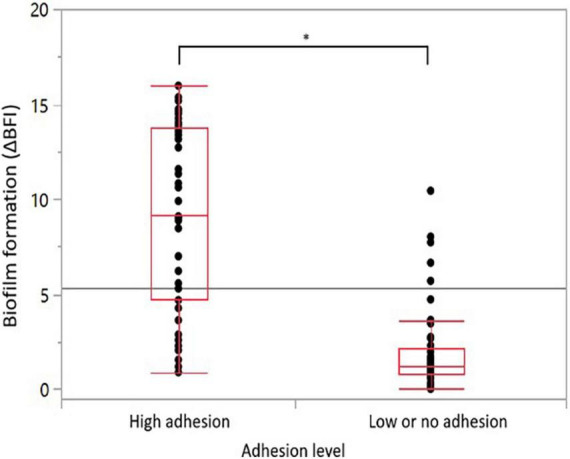
Correlations between adhesion capability and biofilm formation ability. Distribution of the 83 *C. jejuni* strains biofilm formation according to their adhesion capability. Statistical significance was determined using the ANOVA test (*p ≤ 0.05).

Adhesion to an inert surface was measured after a 2-h incubation. The results are expressed as an average of ΔBFI from biological triplicates, which varies from 0.47 to 18.16 among our strain collection (Supplementary Data 4). For biofilm, the ΔBFI average varied from 0.00 to 15.96 after 22 h of incubation under AC (Supplementary Data 4). As expected, the ΔBFI is lower for biofilm as it was developed from only attached cells, which represents a lower concentration of starting culture. As the average ΔBFI for adhesion and biofilm formation assessments were above seven, the closest integer value was chosen to set up categories, i.e., ΔBFI_delimiting_ = 8. The strains were classified into significantly distinct groups: high adhesion (ΔBFI ≥ 8), low or no adhesion (ΔBFI < 8), high biofilm formers (ΔBFI ≥ 8), and low or no biofilm formers (ΔBFI < 8). Overall, a total of 39 (47%) strains were classified with high adhesion, 44 (53%) with low or no adhesion, 27 (33%) strains with significant biofilm formation, and 56 (67%) with no or low biofilm development (Table 3). The positive control, strain Bf, displayed a high adhesion with a ΔBFI average of 17.47 ± 0.72 (*n* = 12) and a high biofilm formation with a ΔBFI average of 13.42 ± 1.54 (*n* = 13) (Supplementary Data 4). The negative control strain, Camp052, developed very low adhesion with a ΔBFI average equal to 1.57 ± 1.57 (*n* = 12) and very low biofilm formation with a ΔBFI average equal to 1.05 ± 0.91 (*n* = 13) (Supplementary Data 4). Statistical analyses indicate that most of the strains were able to form a biofilm when they exhibited a high adhesion (*n* = 25) and strains with no or weak adhesion (*n* = 42) could not form biofilms. The high adhesion was significantly correlated with the high formation of biofilm (ANOVA, *p* < 0.0001) (Figure 3). For risk assessment, when a strain has a strong adhesion, it is 14 times more likely to develop a biofilm (chi-squarest test, *p* < 0.0001). Noticeably, 14 strains were not able to form a biofilm, although they have a high adhesion.

**TABLE 3:** Classification of the 83 *C. jejuni* strains according to adhesion capability to abiotic surfaces and biofilm formation ability.

	Number of strains	Classification
Adhesion capability	44	Low or no adhesion
	39	High adhesion
Biofilm formation ability	56	Low or no biofilm formation
	27	High biofilm formation

### Inter phenotypical correlation tests

Statistical correlation tests were applied between acclimation, oxidative stresses, and adhesion/biofilm responses. Unexpectedly, acclimation to AC shows no significant correlation with oxidative stresses, neither adhesion capability nor biofilm formation. Furthermore, there was no correlation between hyperoxide stress, adhesion to an inert surface, and biofilm formation. In addition, only one weak correlation was established between superoxide stress and the ability of strains to adhere to abiotic surfaces (ANOVA, *p* = 0.0454) (Supplementary Data 5).

### Correlation between epidemiological patterns and phenotypic traits

No significant difference was found between recurrent vs. sporadic strains in their oxidative stress survival responses (*p* > 0.05, Supplementary Data 6) nor in their acclimation to AC capacities (*p* > 0.05). Interestingly, a significant correlation between the recurrent profiles and the adhesion/biofilm ability was observed: the recurrent strains have a significantly higher adhesion/biofilm-forming capacity than the sporadic ones (chi-squarest test, *p* = 0.0266) (Supplementary Data 6).

**FIGURE 4:**
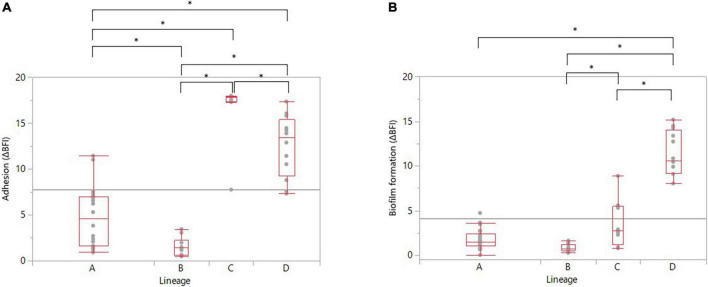
Distribution of **(A)** adherent strains and **(B)** biofilm producers among the 52 recurrent *C. jejuni* strains according to their genomic lineage. Statistical significance was determined using the ANOVA test (*p ≤ 0.05).

The correlation between adhesion and biofilm formation abilities was therefore explored deeper according to the genomic lineages and the different categories of adhesion and biofilm formation. It appeared that each lineage had an adhesion significantly different from the three other lineages (Figure 4A). Overall, lineages C and D had significantly higher adhesion than lineages A and B (ANOVA, *p*-values in Supplementary Data 7). Concerning the distribution of biofilm producers among the strains according to their genomic lineages, it appeared that lineages A and B had a significantly lower capability to develop biofilm (Figure 4B). Surprisingly, lineage C was not classified as a high biofilm producer (ΔBFI median value around 2), although it displayed a significantly higher adhesion ability than lineages A and B (Figure 4). In contrast, lineage D was classified as a high biofilm former with a biofilm-forming capability significantly higher than lineages A, B, and C (ANOVA, *p* < 0.0001 for all three, Supplementary Data 7). There was also a significant difference in biofilm formation between lineages B and C, with lineage B being a less biofilm-producer than lineage C (Figure 4B and Supplementary Data 7).

**FIGURE 5:**
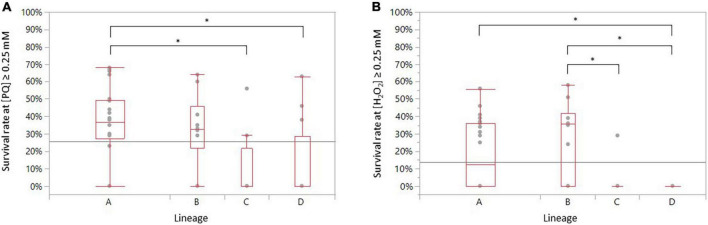
Distribution of the survival rate of the 52 recurrent *C. jejuni* strains exposed to **(A)** PQ and to **(B)** H_2_O_2_ according to their genomic lineage. Statistical significance was determined using the ANOVA test (*p ≤ 0.05).

As lineages are genetically distant from one another, correlations between the different genomic lineages and oxidative stress profiles were also explored. Interestingly, the statistical analyses indicate that strains of lineage A are significantly more resistant to PQ stress than those of lineages C and D (ANOVA, *p* = 0.0393 and *p* = 0.0234, respectively) (Figure 5A). Strains belonging to lineage A survived significantly by 0.25 mM H_2_O_2_ than strains belonging to lineage D (ANOVA, *p* = 0.0151) (Figure 5B). Strains of lineage B demonstrated a significantly higher resistance to a concentration beyond 0.25 mM H_2_O_2_ than strains from lineages C and D (ANOVA, *p* = 0.0171 and *p* = 0.0015, respectively) (Figure 5B).

For acclimation to AC, the data have insufficient power to validate a statistical model because of a lack of representation in each genomic lineage. Consequently, no statistical correlation could be performed between acclimation ability and genomic lineages identified. Nonetheless, a correlation between acclimation to AC and belonging to the different recurrent lineages (chi-squared test, *p* = 0.0135) was observed, which indicates the existence of at least one significant difference between the lineages. It goes toward a trend for acclimation to aerobiosis for lineages A and D. To strengthen this result and increase the reliability, the number of strains in some groups has to be statistically more representative.

### Distribution of the fluoroquinolone resistance according to the genomic lineages

A high proportion of strains belonging to lineages A (CC ST-21), B (CC ST-257), C (CC ST-464), and D (CC ST-21) were predicted resistant to FQ (Supplementary Data 8). No correlation between the resistance to FQ and the genomic lineages was established (chi-squared test, *p* > 0.05). Therefore, fluoroquinolone resistance prediction could not discriminate different phenotypical behaviors among the genomic lineages.

### Functional genomic approach of lineages using Venn diagram

**FIGURE 6:**
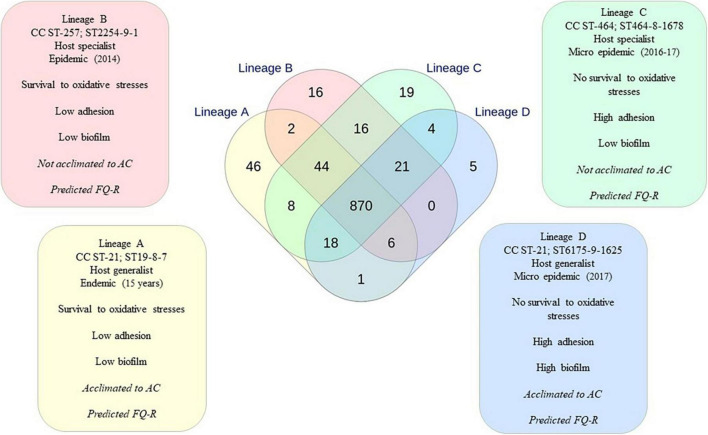
Metaphenotypical profile linked to each recurrent genomic lineage and the number of specific targets related to them. Relationships between the loci identified in the four lineages and specific targets were obtained from the Venn diagram performed previously using wgMLST INNUENDO typing scheme (Figure 3, Nennig et al., 2021). Phenotypes about AAC are mentioned in italic, as they need to be consolidated. Phenotypes about fluoroquinolone resistance (FQ-R) are mentioned in italic, as they are only *in silico* predictions. Factors identified as potentially linked to the phenotypical responses are listed in a colored box.

According to our results, all the characterized phenotypical behaviors related to persistence and transmission (oxidative stress responses, acclimation to AC, and biofilm development) were independent. However, correlation analyses indicated that specific behavior combining independent phenotypes could be attributed to each recurrent lineage. This phenotype combination specific to each lineage was defined as a metaphenotype (Figure 6). For instance, the metaphenotype of lineage A is composed of survival against oxidative stresses and no or low adhesion and biofilm formation, with a tendency for the acclimation to AC. Our study indicated that within the CC ST-21, two different metaphenotypes could be distinguished: the one described for lineage A and the one described for lineage D (Figure 6). Using the INNUENDO typing classification, the Venn diagram resulting from the comparison of the 2,795 targets of the pan-genome (wg) MLST was identical to the one obtained previously (Nennig et al., 2021), indicating that the environmental strains and strain BF did not change the accessory genome. Therefore, the shared targets identified previously for lineages A, B, C, and D were used to compare the recurrent genomic lineages.

In this study, the inferred function was performed from the targets involved in each metaphenotype identified. As lineage A displayed an endemic pattern, its 46 unique targets were examined. The specific targets of lineage A encode for enzymes such as a DNA primase (encoded by *dnaG*), a peptidase, or different methyltransferases. Moreover, five proteins are involved in diverse biosynthesis pathways, and eight others are originated from phages, including phage integrase, phage protein, phage gp6-like head-tail connector protein encoded by *intA*. In addition, 19 proteins are conserved with unknown functions (Supplementary Data 9).

The difference in phenotype characterization between lineages A and B relies on reinforcement of data about acclimation to AC. As two different targets were identified between lineages A and B (Supplementary Data 9), this indicates that the open reading frames (ORF) found in these two targets with unknown functions (*Cj1089c* and *Cj0243c*) could contribute to the oxidative stress survival, to the absence of a high adhesion or a high biofilm formation. Further investigations are required on these genes to elucidate their role in *C. jejuni*.

Concerning the oxidative stress response, a significant resistant trend was identified for lineages A and B while a significant susceptible pattern was observed for lineages C and D. If the oxidative stress susceptibility phenotype results from the same cellular mechanism regardless of the lineages, then targets shared by lineages A and B but not by lineages C and D would help to explain the oxidative stress resistance phenotype and targets shared by lineages C and D would contribute to explain the oxidative stress sensitivity phenotype. Only two targets were identified to be common to lineages A and B (Supplementary Data 9) but not to lineages C and D, and four targets were identified to be common to lineages C and D but not to lineages A and B (Supplementary Data 9). In the latter targets, the *rfbE* encoding for RfbE protein is a sugar-nucleotide epimerase-dehydratase catalyzing the cytidine diphosphate-3,6-dideoxy-D-glucose to give the cytidine diphosphate-3,6-dideoxy-D-mannose. The TssB and TssC proteins compose the contractile sheath of the T6SS (Supplementary Data 9), and the last target is composed of a protein with an unknown function. As phenotype characterization indicated a difference in the ability to develop a biofilm between lineages C and D, the shared targets between these two lineages could contribute to explain the adhesion capacity but not the one for biofilm development. Furthermore, the 19 unique targets in lineage C might contribute to enhance adhesion or to prevent biofilm development while the five unique targets for lineage D might be involved in biofilm development (Supplementary Data 9). Among the unique targets of lineage C, the *mshL* gene encodes for the pilus biogenesis protein MshL and a DNA repressor were identified (Supplementary Data 9). In addition, three other targets related to genes encoding for T6SS elements (*tssG*, *hcpA*, and the ImpG/VasA protein) were also identified to be unique. However, the blastP analyses on data extracted from the NCBI RefSeq genome database revealed that these proteins were also present in lineage D. The difference concerns the lower homology between strains of lineage C and D with 64, 62, and 89% of protein identity, respectively. This difference might result from primary to quaternary structure differences in the resulting proteins. Concerning lineage D, two of the five unique targets could be identified as RloA and a serine dehydratase (Supplementary Data 9). RloA is an ATPase, while the serine dehydratase is involved in the formation of glucose from non-carbohydrate precursors, such as pyruvate, amino acids, and glycerol. Further analyses on T6SS components, including gene synteny prospect from the sequencing data, reveal that 12 out of 13 core components were present in lineages C and D (apart from homology differences) but not in lineages A and B. Their identification using the UNIPROT reference through the blastP analyses indicated 89–100% identity. For instance, HcpA amino acid sequences were 100% identical among the strains belonging to lineages C and D (Supplementary Data 10). As previously observed in *C. jejuni* (Robinson et al., 2021), the absence of ClpV, also called TssH, was observed in these strains leading to an incomplete T6SS. In parallel, all the genes corresponding to T6SS core components present in lineage C and D were not detected in lineages A and B.

## Discussion

*Campylobacter jejuni* is known for its genome plasticity which results from a high DNA mutation rate (3.4 × 10^–6^ substitutions per site per year) and frequent homologous recombinations due to a facilitated horizontal gene transfer (Calland et al., 2021). However, our previous study highlighted the presence of monomorphic lineages regularly infecting humans with a broad distribution over time and sources, suggesting persistence in reservoirs and in the environment (Nennig et al., 2021). From a representative collection of 83 *C. jejuni* strains isolated in Luxembourg from various sources (human, mammals, poultry, and environment) and different spreading profiles (endemic, epidemic, and sporadic), the classification of genomic lineages A, B, C, and D could be reproduced using the Oxford typing scheme, as described previously (Nennig et al., 2021). This analysis on a more representative panel of strains, including strains isolated from the environment, confirmed the spatiotemporal distribution of more stable genomes over years. Lineages were related to endemic, micro-epidemic, and emerging profiles. This classification is generally similar to the one of Sheppard et al. (2014) using MLST databases and host attribution. However, it is more precise as the host-generalist CC ST-21 could be divided into two distinct lineages (A and D). Considering the presence of monomorphic populations in *C. jejuni*, we hypothesized that strains from the same lineage might share adaptive behaviors. Frequent human infections by the strains of these lineages would suggest that these strains developed joint strategies contributing to the emergence and the persistence of *C. jejuni* genotypes throughout the food chain and environment. To characterize phenotypes related to the survival and the transmission of *C. jejuni*, several different tests were selected, covering the adaptation potentialities of *C. jejuni* based on previous studies on the highest risks of transmission. A correlation was found between adhesion and biofilm formation and between superoxide and hyperoxide stresses; however, no correlation was found between the different phenotypes tested. The absence of interdependency could not segregate the genomic lineages according to only one phenotype, indicating that the cellular and molecular mechanisms underlying these phenotypes are different. However, our study revealed that a genomic lineage could be characterized by a specific combination of independent phenotypes, referred to here as a metaphenotype. The analyses of the different metaphenotypes were performed according to the unique or lineage-shared targets as revealed by the Venn diagram built up from wgMLST INNUENDO data to compare targets of the pan-genome of the *C. jejuni* strain collection. When possible, open reading frames (ORFs) included in each sequence target were identified, and the function was attributed using functional genomic approaches. The benefits of AD comparison encompass the gene-to-gene comparison, gene polymorphisms as well as single nucleotide polymorphisms (SNPs). Nonetheless, the undefined targets and/or the great proportion of proteins with unknown functions (35/117 for unique targets and targets shared between two lineages) in *C. jejuni* limit the issue.

Several studies have investigated how to score the impact of oxidative stresses on bacterial survival by implementing different techniques (Hwang et al., 2011; Oh et al., 2015a,b; Rodrigues et al., 2015; Dai et al., 2017; Mouftah et al., 2021). In our study, PQ and H_2_O_2_ were used as prooxidants to generate oxidative stresses on *C. jejuni*. PQ simulates the superoxide stress while H_2_O_2_ induces the hyperoxide stress. These two stresses were investigated as *C. jejuni* possesses a range of enzymes involved in oxidative stress defense with detoxification mechanisms stimulated by PQ and H_2_O_2_, including SodB, KatA, AhpC, and Tpx (Pesci et al., 1994; Grant and Park, 1995; Day et al., 2000; Garénaux et al., 2008; Atack and Kelly, 2009). The responses to oxidative stresses were well distributed among our strain collection and a correlation between H_2_O_2_ and PQ survival at a concentration above 0.25 mM was demonstrated. These results are consistent with previous reports, indicating different susceptibilities of *C. jejuni* to both oxidant agents (Rodrigues et al., 2015). It was demonstrated that PerR, Fur, and CosR regulate SodB, AhpC, and KatA in *C. jejuni* (Atack and Kelly, 2009; Hwang et al., 2011, 2012). CosR dowregulates SodB; however, in the presence of PQ, the CosR expression level is reduced (Hwang et al., 2011). Thus, the reduction of CosR expression in the presence of PQ could decrease SodB repression, leading to an accumulation of H_2_O_2_, which could enhance the sensitivity of the strains to oxidative stress and correlatively could decrease their survival. This could explain why some strains did not survive the oxidative stress generated by PQ but survived in H_2_O_2_ assays. In addition, the accumulation of H_2_O_2_ is possibly higher with PQ assay as it is a redox cycler which causes a more sustained oxidative stress. However, a better survival in PQ assays could result also in an upregulation of KatA which actively detoxifies the accumulated H_2_O_2_ (Garénaux et al., 2008). Overall, the survival against oxidative stresses did not appear to discriminate the long-term occurrence of the strains from sporadic ones. However, lineages A and B are characterized by a marked survival ability against oxidative stresses in contrast to lineages C and D that did not significantly survive oxidative stresses. This is in accordance with previous results showing that host generalist clonal complexes have a greater tolerance to oxidative stress (Mouftah et al., 2021).

*Campylobacter jejuni* is a strict microaerophilic pathogen requiring a low concentration of dioxygen (∼5–6%) for optimal growth; therefore, the main stress it encounters in the extra-intestinal environment is mainly the atmospheric concentration of O_2_ (Kaakoush et al., 2007; Macé et al., 2015). Reduced susceptibility or acclimation ability to AC may enable greater environmental persistence, thus increasing the risk of transmission between prospective hosts. As described by Oh et al. (2018), aerotolerance contributes to the survival of *C. jejuni* in chilled chickens and its prevalence in human clinical cases. Previous studies have investigated *C. jejuni* aerotolerance capacity (Oh et al., 2018; Kim et al., 2019) and the mechanism of regulation of aerotolerance (Baillon et al., 1999; Atack et al., 2008; Handley et al., 2015) demonstrating a high prevalence of hyper-aerotolerant strains in poultry (raw chicken and duck meat). Through transcriptomic and proteomic assays, studies highlighted that many proteins involved in host colonization (e.g., PorA and CadF) were more abundant at lower oxygen availability (1.8% O_2_). A coordinated response of oxidative stress protection enzymes (e.g., SodB, AhpC, Tpx, and TrB) and Fe-S cluster biogenesis proteins were observed in oxidative stress conditions (i.e., increased level of O_2_ from 5 to 17.5%) (Guccione et al., 2017). Rodrigues et al. (2016) also reported higher transcript levels for Tpx and SodB in AC than in MAC using proteomic, gene expression, and enzymatic approaches. However, most of the studies were based on the detection of survival under harmful conditions. Few strains of *C. jejuni* and *C. coli* have been described in the literature as being able to acclimate and to multiply in AC (Rodrigues et al., 2015; O’Kane and Connerton, 2017). Bacterial acclimation is defined as the increasing capability to resist and multiply under several consecutive exposures to harmful conditions. To have a broader view on this unique fitness, the collection of *C. jejuni* was screened for acclimation to AC. Surprisingly, the results indicate that more than half of the strains could acclimate to AC on solid media. Among them, 12% were even able to grow on non-selective media under AC. For most of the isolates, aerobic growth was low during the first sub-culture but significantly enhanced after at least two subsequent passages. With the implementation of an intermediate step (growth on selective charcoal-based medium) acting as an oxygen-reducer, we were able to segregate the strains with higher acclimation capability (AAC) from those with lower acclimation ability (SAAC). Altogether, a minimum of three consecutive passages were required to ensure the reproducibility of the data. Very recently, the same conclusions were observed on strains of *C. jejuni* (Shagieva et al., 2021). The authors focused on the study of *C. jejuni* harvested from wastewater and intracellular growth preservation into amoebas. In addition, our study reveals that this capability to acclimate to AC is not related to the isolation source as strains from both clinical, animal, and environment sources could develop this ability. In our study, the acclimated colonies lost the ability to re-grow in AC directly after freeze storage. The reproduction of acclimation phenomenon for those strains could be obtained only after a first culture under MAC followed by at least two passages under AC. The comparison of the core genome sequences from our WGS data before and after acclimation to AC indicated the absence of genome alterations in four strains over the six tested. Only one allelic difference was observed for Camp022 and Camp098 resulting in an amino acid transition in the *Cj0276* locus, the gene *mreB*. MreB is involved in the shape transition from rod to coccoid shape in *C. jejuni* (Chiu et al., 2008). As this allelic difference does not appear in the other acclimated strains tested, we assumed that it is not a determinant for acclimation to AC mechanism. This result indicates that the acclimation to AC fitness is likely not governed at the genetic sequence level. Epigenetics regulation could be one of the hypotheses to explain this transient acclimation mechanism. The emergence of epigenetic lineages enables the adaptation of bacterial populations to harsh environments and moderates their behavior (Beaulaurier et al., 2019; Sánchez-Romero and Casadesús, 2020). For instance, a study revealed phenotypical evidences supporting the hypothesis that *C. jejuni* methyltransferases play a regulatory role in phenotypes, such as motility and host cell adhesion (Kim et al., 2008). Mou et al. (2015) detected hypo and hyper-methylated regions in *C. jejuni* genomes, and they demonstrated that restriction-modifications (RM) activities may play a role in the gene expression which might be correlated with the hypervirulence phenotype of the SA clone by comparing the methylome profiles of NCTC 11168, 81–176, and the SA clone (Mou et al., 2015). Indeed, studies revealed that they have a putative role in regulating several virulence genes (e.g., a flagella gene *flhB* and a RNA polymerase sigma factor *rpoN*) (Ghatak et al., 2020). Thus, the combined action of the methyltransferases and the RM system identified in our genomic lineages could be an explanation for the enhanced diversity of phenotypical responses we observed. The presence of proteins related to methyltransferases (*hsdM* gene) and to type I restriction-modification system (subunit S encoded by the *hsdS* gene) among the unique targets identified in lineage A fits this hypothesis. Given the occurrence of the metaphenotype and the relevance of the acclimation process in *C. jejuni*, this should be further investigated to determine whether this tendency could result from a clonal expansion that is sustained over time.

Currently used methods to study biofilms include microtiter plates to screen the biofilm formation capacity or to screen anti-biofilm molecules based on biofilm biomass (Azeredo et al., 2017). As crystal violet staining is not well adapted to *Campylobacter* detection because of a lower sensitivity (Sulaeman et al., 2010; Svensson et al., 2014), we selected the Biofilm Ring test^®^ to detect adhesion and biofilm formation in microtiter plates, due to its high-throughput and ease of use (Chavant et al., 2007; Azeredo et al., 2017). A study comparing bacterial adhesion with the BioFilm Control and reverse ELISA methods has previously validated the use of the BioFilm Ring test^®^ for *Campylobacter* (Sulaeman et al., 2010). Here, we developed a procedure to assess biofilm formation and determined parameters for the starting culture, growth conditions, and incubation time. The results of adhesion and biofilm formation tests indicated a wide diversity in adaptive responses among our collection going from low/no adhesion to high adhesion and low/no biofilm producers to high biofilm-producers. Growing in AC was a factor of success for biofilm formation of *C. jejuni* (Gunther and Chen, 2009; Sulaeman et al., 2010; Turonova et al., 2015; Teh et al., 2019), which was confirmed here. As expected, a significant correlation was established between the adhesion to abiotic surfaces and biofilm formation. For risk assessment, it was established that a highly adherent *C. jejuni* strain is 14 times more likely to develop a biofilm. A correlation between adhesion to abiotic surfaces and biofilm development has been demonstrated in other bacterial species, such as cyanobacteria (Faria et al., 2021). However, the wide diversity of the responses is more specific to *C. jejuni*. For instance, less diversity was observed for adhesion by *Listeria monocytogenes* as all the strains were able to attach to abiotic surfaces (Tresse et al., 2007). This study revealed a significant correlation between adhesion to an inert surface and biofilm development for the strains with a recurrent profile. The strains of lineages A and B were not able to adhere and form biofilms while those of lineages C and D were more adherent-proficient genotypes. However, the proven ability to form biofilms was observed only for lineage D. Strains of lineage C were significantly able to adhere firmly but did not produce strong biofilm. The absence of a strong biofilm formation was previously described for the strain NCTC 11168 compared to strain 81–176 (Turonova et al., 2015). The authors observed a dense biofilm showing layers of attached cells without any pores or channels to ensure nutrient and gas exchanges, which consequently hindered the survival of the biofilm. This led to the hypothesis that the biofilm could not enter the maturation phase. A two-component regulator, CosR, or enzymes, such as AhpC, appear to play a role in this biofilm maturation step (Oh and Jeon, 2014; Turonova et al., 2015).

The presence of 12 out of 13 core components of the T6SS was detected only in the metaphenotypes of lineages C and D, with three components showing less sequence homology between the two lineages. The T6SS is a secretion system that enables the translocation of effector proteins to diverse prokaryotic cells and thus participates in inter-bacterial competition and pathogenesis (Zoued et al., 2014). Studies examining the prevalence of the T6SS in *C. jejuni* over Europe indicated a large variation in prevalence. In 2014, approximately 3% of chicken isolates from the United Kingdom were T6SS-positive, while 28.8% of similar isolates were T6SS-positive in Northern Ireland in 2015 (Harrison et al., 2014; Corcionivoschi et al., 2015). The core component TssH (ClpV) was not found in any *C. jejuni* strain in our collection, which is in accordance with previous results reported on *C. jejuni* harboring the T6SS (Liaw et al., 2019; Robinson et al., 2021). Compared to the T6SS assemblage machinery in other bacteria, this protein is an ATPase responsible for the disassembly of the contracted sheath components. Despite the absence of ClpV in *C. jejuni*, the presence of a functional T6SS was demonstrated in *C. jejuni* and in other organisms (e.g., *Helicobacter hepaticus* or *Salmonella* Typhimurium) (Lertpiriyapong et al., 2012; Sana et al., 2016; Kanwal et al., 2019). An alternative mechanism for the sheath disassembly or the existence of a ClpV-like ATPase encoded near the T6SS cluster might exist (Liaw et al., 2019). Interestingly, one of the unique targets identified in lineage D was an ATPase called RloA. This enzyme was detected near the *vgrG* gene, encoding one of the T6SS core components. The RloA protein is a member of the AAA+ superfamily, which is composed of proteins involved in a variety of different functions, such as protein unfolding and degradation (Snider et al., 2008). The presence of a ClpV-like ATPase was also mentioned by Robinson et al. (2021) as a serious potential alternative to ClpV (Robinson et al., 2021). This possibility suggests the presence of a complete and functional T6SS in metaphenotype of lineage D but not in the other metaphenotypes. In *C. jejuni*, the T6SS biological functions have been associated with host colonization, host cell adhesion, and invasion, contributing to oxidative stress defense (Lertpiriyapong et al., 2012; Liaw et al., 2019). The secreted effectors display a wide range of functions (nucleases, lipases, or pore-forming activities), and their injection into competing bacteria promotes the fitness of T6SS-positive strains in polymicrobial environments like the gut ecosystem, facilitating their survival (Jiang et al., 2016; Coulthurst, 2019; Jana et al., 2019; Fridman et al., 2020; Wood et al., 2020). Furthermore, it has been demonstrated that the T6SS could enhance biofilm formation in *Acinetobacter baumannii* or be involved in biofilm maturation in *Pseudomonas fluorescens* (Gallique et al., 2017; Kim et al., 2017). This may therefore explain strain NCTC 11168’s inability to enter the biofilm maturation phase (Turonova et al., 2015) and might explain why some strains can adhere but cannot form a biofilm. As the metaphenotype of lineage D is characterized by the capability to form a biofilm, in contrast to the metaphenotype of lineage C, and as this lineage D possesses a potential complete T6SS with RloA, then this secretion system might be connected with biofilm growth and maturation in *C. jejuni.* We stipulate this to be a potential molecular marker to identify strains able to develop a biofilm among *C. jejuni* population.

The clonal expansion model of lineage A could be explained by the acquisition of unique targets related to phage. The bacteria-phage coevolution is known as a driver of ecological and evolutionary processes in microbial communities (Koskella and Brockhurst, 2014). Previously, *C. jejuni* isolates harboring CJIE1 prophage were linked to specific phenotypic behaviors, including invasion abilities (Clark et al., 2016). Furthermore, another unique target identified in lineage A belongs to type II toxin-antitoxin (TA) system of the PemIK/MazEF family toxin. MazEF is a TA module widely distributed among many bacterial species, such as *Escherichia coli, Staphylococcus* genus, or *Campylobacter* spp. (Yan et al., 2012; Bukowski et al., 2017). TA modules consist of a pair of antagonistic genes that encode for a stable toxin and an unstable antitoxin. Recent studies have demonstrated that they play a central role in bacterial persistence (Page and Peti, 2016). Thus, these genetic determinants could play a role in the long-term occurrence of the strain in lineage A, which has been endemic for at least a 15-year period in Luxembourg.

In addition, predicted resistance to fluoroquinolones inferred from sequence data was performed to complete the phenotypic profile of the strains. The correlation between a single mutation in the *gyr*A gene and the phenotypic resistance of the strains to ciprofloxacin and nalidixic acid has been shown to be statistically significant with or without the synergistic action of multidrug pump efflux (Yao et al., 2016; Elhadidy et al., 2020). The AMR pattern of *C. jejuni* influences its survival by providing an improved biological fitness. Good et al. (2019) demonstrated that multi-drug resistant *C. jejuni* strains were among the longest surviving isolates to antibiotherapy (Luo et al., 2005; González and Hänninen, 2012; Good et al., 2019). Furthermore, the ability of FQ-resistant strains to persist in the environment (e.g., in poultry production), after the removal of the selective pressure, was also demonstrated (Price et al., 2007). With the increase of AMRs among isolates from clinical, veterinary, and environmental sources, *C. jejuni* was classified as a serious antimicrobial-resistant threat. Several studies were performed to define the frequency of resistance among specific phylogenetic lineages. Indeed, even though AMRs were distributed among isolates belonging to relatively distant lineages, indicating a widespread dispersal, Wimalarathna et al. (2013) showed evidence of clustering of resistance phenotypes within lineages, pointing out local expansion of resistant strains (Wimalarathna et al., 2013). A study performed in Slovenia determined antibiotic resistance and MLST profiles of 52 *C. jejuni* isolates from diverse sources (human, animal, chicken meat, and water). They showed a high incidence of FQ-resistant strains within CC ST-21 due to clonal spreading rather than high genetic plasticity (Kovač et al., 2014). This high prevalence in drug-resistance within CC ST-21 is in accordance with our results as 32 out of 34 strains, belonging to lineages A and D, belong to CC ST-21 and are FQ-resistant. An association study between ST and quinolone resistance was also performed on strains belonging to CC ST-464 (lineage C in our study) (Cody et al., 2012). The increase in their relative incidence in Europe, as well as their association with ciprofloxacin resistance, was also confirmed by Kittl et al. (2013). We observed the same phenomenon in our results, as all the strains belonging to CC ST-464 were FQ-resistant. This lineage was also reported in a study performed in United States, where patients reporting travel history were more likely to be linked to infections caused by this ST (Cha et al., 2016). Given the AMR prediction and the high percentage of resistance to FQ within the selected panel, our results confirm the persistence of FQ-resistant lineages and their dissemination outside of Europe over the years.

According to our analyses, a combination of independent biological responses was specific to each lineage. These data suggest that a combination of different phenotypical abilities, the metaphenotype, may contribute to the adaptation and survival of these monomorphic lineages over time and from various sources. These results also indicate the existence of a selective pressure behind the emergence and persistence of the most adapted and resistant lineages to different environmental stresses. Survival strategies of bacterial pathogens outside the host could lead to the selection of specific genetic profiles and changes in gene deregulation. Reversible switches in the expression of genes (phase variation) have been reported in *C. jejuni*, leading to numerous phenotypes (Bayliss et al., 2012). Within the unique and shared targets identified in lineages A, B, and C, specific methyltransferases were found in each of them. These enzymes are involved in DNA methylation, which regulates the reversible switching (phase variation) of gene expression in many bacterial species, a mechanism that produces phenotypic variations. The emergence of epigenetic lineages allows the adaptation of bacterial communities to challenging environments and to moderating the behavior of pathogenic agents (Sánchez-Romero and Casadesús, 2020). For instance, DNA methylation could be an avenue to consider the acclimation to the AC trend of *C. jejuni* strains. On the other hand, the emergence of clone expansion can hardly be explained by a single biological advantage, as this phenomenon is rather multifactorial. Indeed, the different phenotypic responses did not exhibit interdependence, and lineages were rather characterized by a metaphenotype. This work highlighted some potential molecular markers, gave some avenues to understand better the behavior of *C. jejuni*, and contributed to appreciate the risk about controlling the spreading of these lineages, which are responsible for half of the human infections in Luxembourg. The survival and persistence of *C. jejuni* in the environment enhances the effectiveness of its transmission to humans in terms of risk exposure and thus reinforces a public health risk. Further experimentations and analyses might focus on the analysis of non-coding regions and on metaphenotypes related to lineages exhibiting a clonal expansion and associated with human infections.

## Data availability statement

The datasets presented in this study can be found in online repositories. The names of the repository/repositories and accession number(s) can be found in the article/Supplementary material.

## Author contributions

MN carried out the phenotypical and functional genomic analyses and wrote the initial draft of the manuscript. MN developed the protocol for biofilm formation with CR and EL, who were involved in data acquisition for this test. OT and CR conceived, designed, and oversaw the study. OT helped to optimize the protocols. AC performed statistical analyses. OT and TB brought their expert critical point of view on the current work. TB provided materials and analysis tools for adhesion and biofilm formation tests. All authors have critically reviewed and approved the published version of the manuscript.

## Footnotes

1
https://mage.genoscope.cns.fr/

2
https://www.geneious.com/

3
https://www.softpedia.com/get/Science-CAD/BioEdit.shtml

## Abbreviations

AAC: acclimated to aerobic conditions
AC: aerobic conditions
AD: allele difference
BFI: biofilm index
CC: clonal complex
MAC: microaerobic conditions
NAAC: non-acclimated to aerobic conditions
OD: optical density
PQ: paraquat
ROS: reactive oxygen species
ST: sequence type
T6SS: type VI secretion system.
