# Supplementary material for: Metaphenotypes associated with recurrent genomic lineages of Campylobacter jejuni responsible for human infections in Luxembourg
Source: Front Microbiol. 2022 Sep 7;13:901192. doi: 10.3389/fmicb.2022.901192 (PMC9490421; doi:10.3389/fmicb.2022.901192)
Supplement: Supplementary file 1 [file Presentation_1.PPTX]

## Slide 1
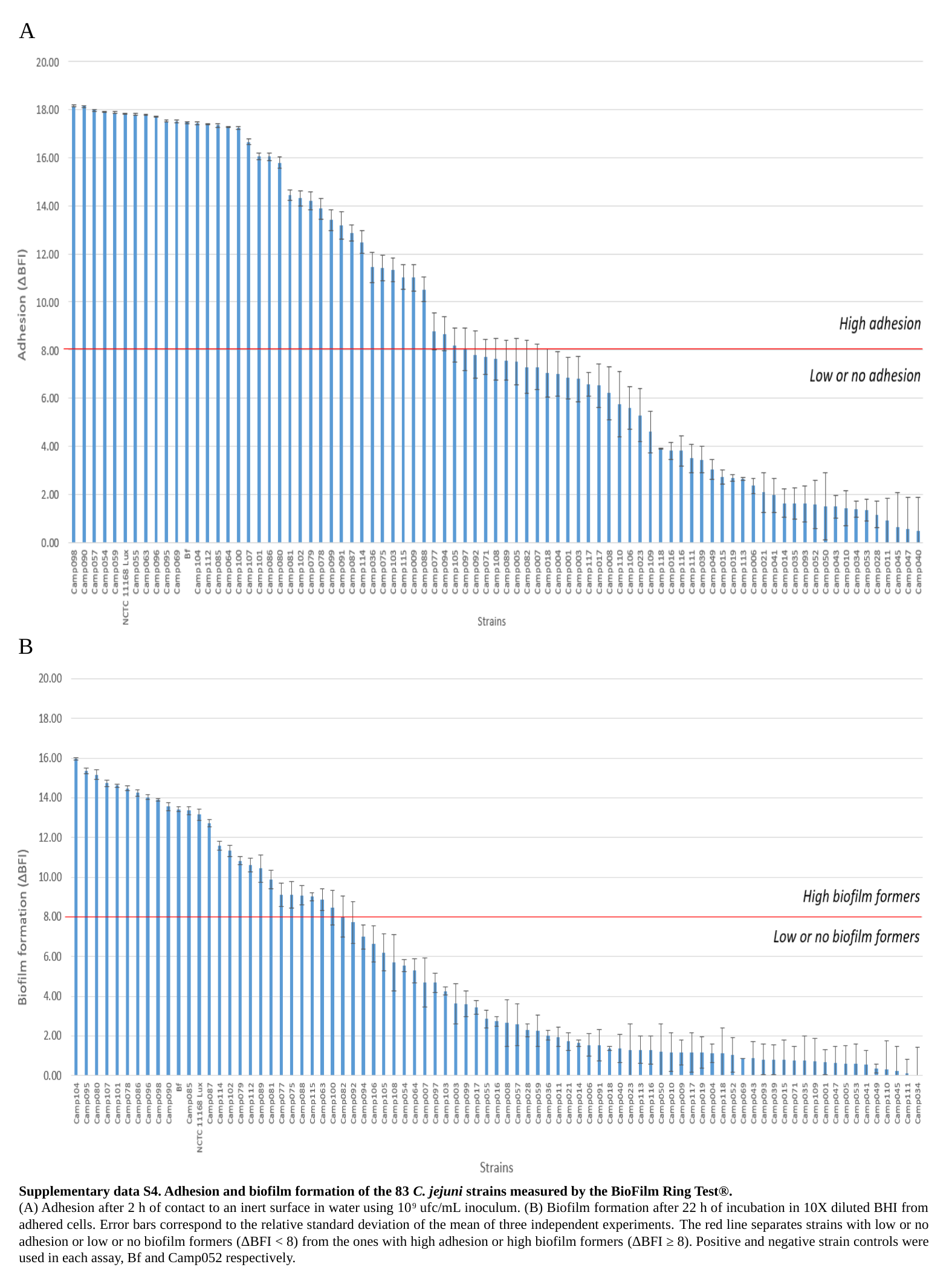

A
B
Supplementary data S4. Adhesion and biofilm formation of the 83 C. jejuni strains measured by the BioFilm Ring Test®.
(A) Adhesion after 2 h of contact to an inert surface in water using 109 ufc/mL inoculum. (B) Biofilm formation after 22 h of incubation in 10X diluted BHI from adhered cells. Error bars correspond to the relative standard deviation of the mean of three independent experiments. The red line separates strains with low or no adhesion or low or no biofilm formers (ΔBFI < 8) from the ones with high adhesion or high biofilm formers (ΔBFI ≥ 8). Positive and negative strain controls were used in each assay, Bf and Camp052 respectively.
