# Supplementary material for: Metaphenotypes associated with recurrent genomic lineages of Campylobacter jejuni responsible for human infections in Luxembourg
Source: Front Microbiol. 2022 Sep 7;13:901192. doi: 10.3389/fmicb.2022.901192 (PMC9490421; doi:10.3389/fmicb.2022.901192)
Supplement: Supplementary file 2 [file Presentation_2.PPTX]

## Slide 1
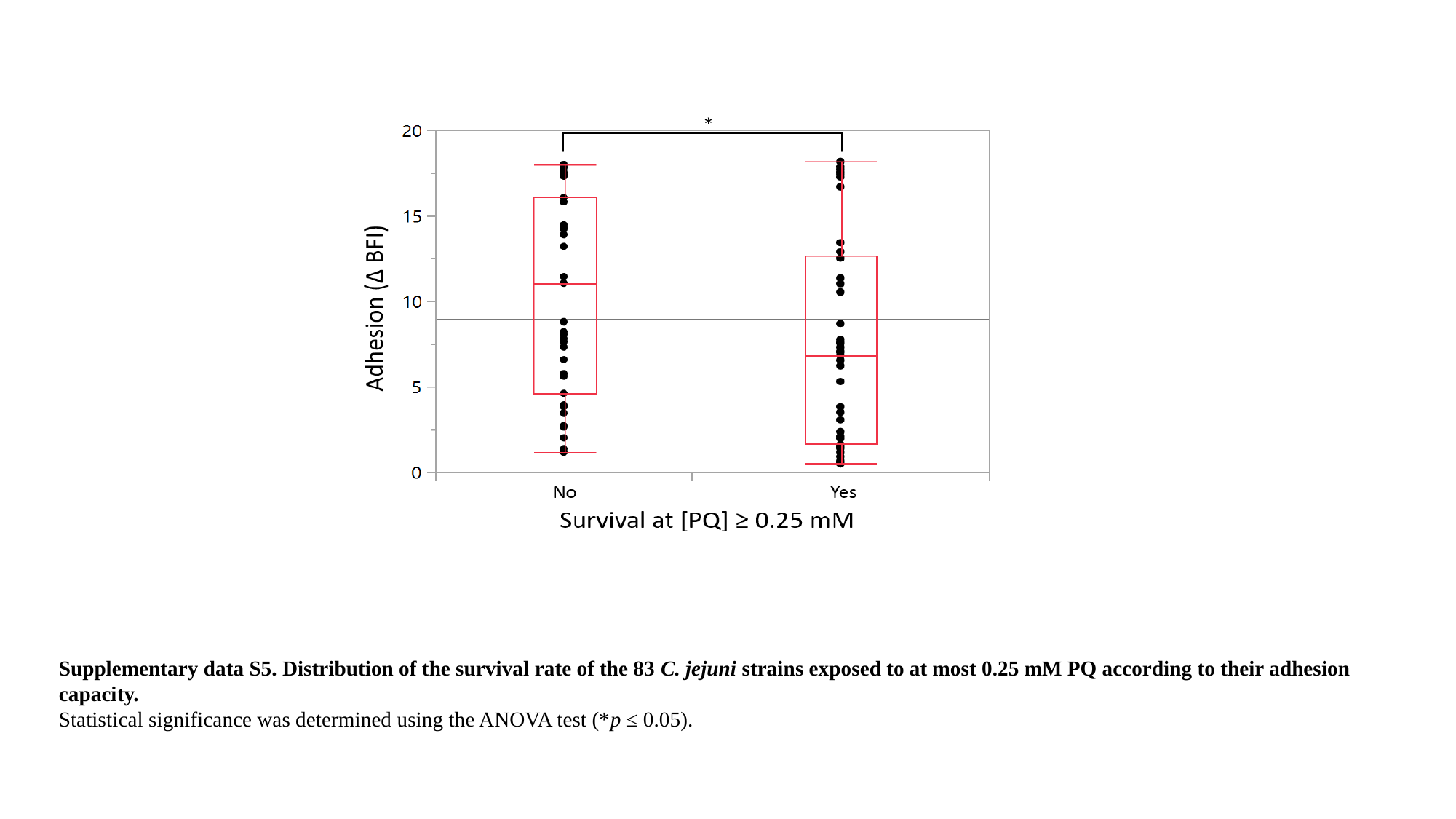

Supplementary data S5. Distribution of the survival rate of the 83 C. jejuni strains exposed to at most 0.25 mM PQ according to their adhesion capacity.
Statistical significance was determined using the ANOVA test (*p ≤ 0.05).
