# Supplementary material for: Metaphenotypes associated with recurrent genomic lineages of Campylobacter jejuni responsible for human infections in Luxembourg
Source: Front Microbiol. 2022 Sep 7;13:901192. doi: 10.3389/fmicb.2022.901192 (PMC9490421; doi:10.3389/fmicb.2022.901192)
Supplement: Supplementary file 3 [file Presentation_3.PPTX]

## Slide 1
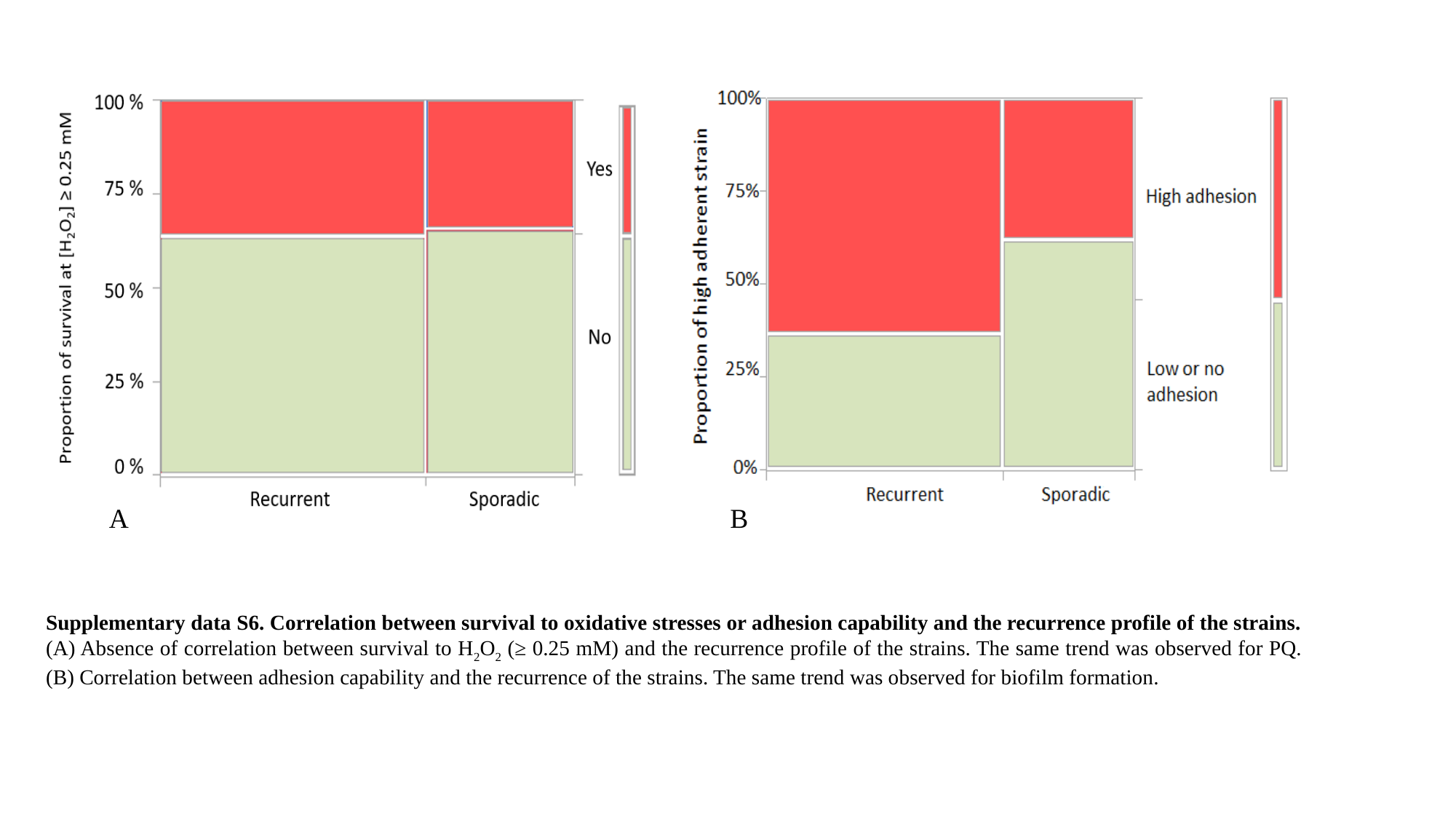

A
B
Supplementary data S6. Correlation between survival to oxidative stresses or adhesion capability and the recurrence profile of the strains.
(A) Absence of correlation between survival to H2O2 (≥ 0.25 mM) and the recurrence profile of the strains. The same trend was observed for PQ. (B) Correlation between adhesion capability and the recurrence of the strains. The same trend was observed for biofilm formation.
