# Supplementary material for: Metaphenotypes associated with recurrent genomic lineages of Campylobacter jejuni responsible for human infections in Luxembourg
Source: Front Microbiol. 2022 Sep 7;13:901192. doi: 10.3389/fmicb.2022.901192 (PMC9490421; doi:10.3389/fmicb.2022.901192)
Supplement: Supplementary file 4 [file Presentation_4.PPTX]

## Slide 1
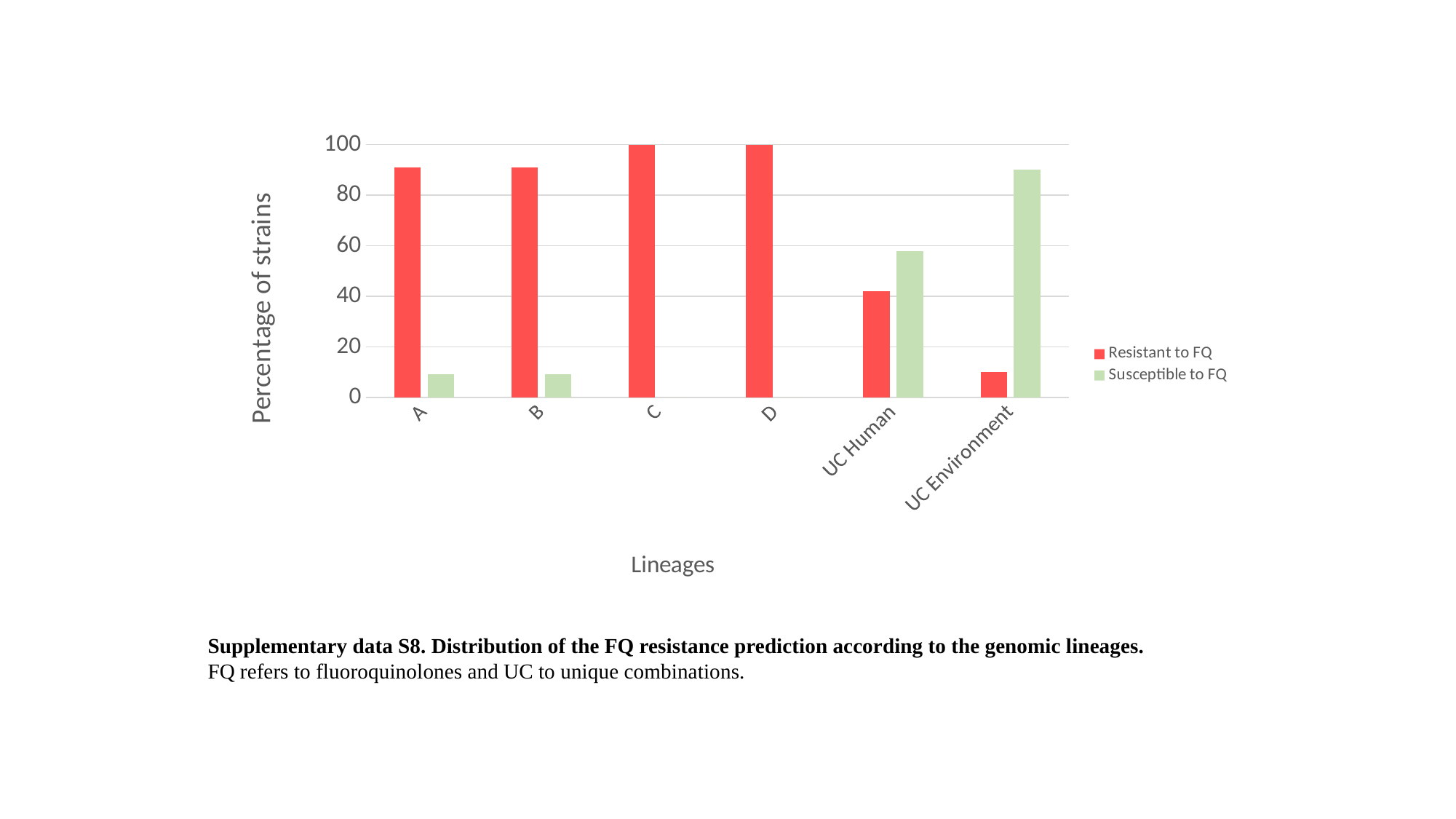

### Chart
| Category | Resistant to FQ | Susceptible to FQ |
|---|---|---|
| A | 90.9090909090909 | 9.090909090909092 |
| B | 90.9090909090909 | 9.090909090909092 |
| C | 100.0 | 0.0 |
| D | 100.0 | 0.0 |
| UC Human | 42.10526315789474 | 57.89473684210526 |
| UC Environment | 10.0 | 90.0 |Supplementary data S8. Distribution of the FQ resistance prediction according to the genomic lineages.
FQ refers to fluoroquinolones and UC to unique combinations.
