# Supplementary material for: Metaphenotypes associated with recurrent genomic lineages of Campylobacter jejuni responsible for human infections in Luxembourg
Source: Front Microbiol. 2022 Sep 7;13:901192. doi: 10.3389/fmicb.2022.901192 (PMC9490421; doi:10.3389/fmicb.2022.901192)
Supplement: Supplementary file 5 [file Presentation_5.PPTX]

## Slide 1
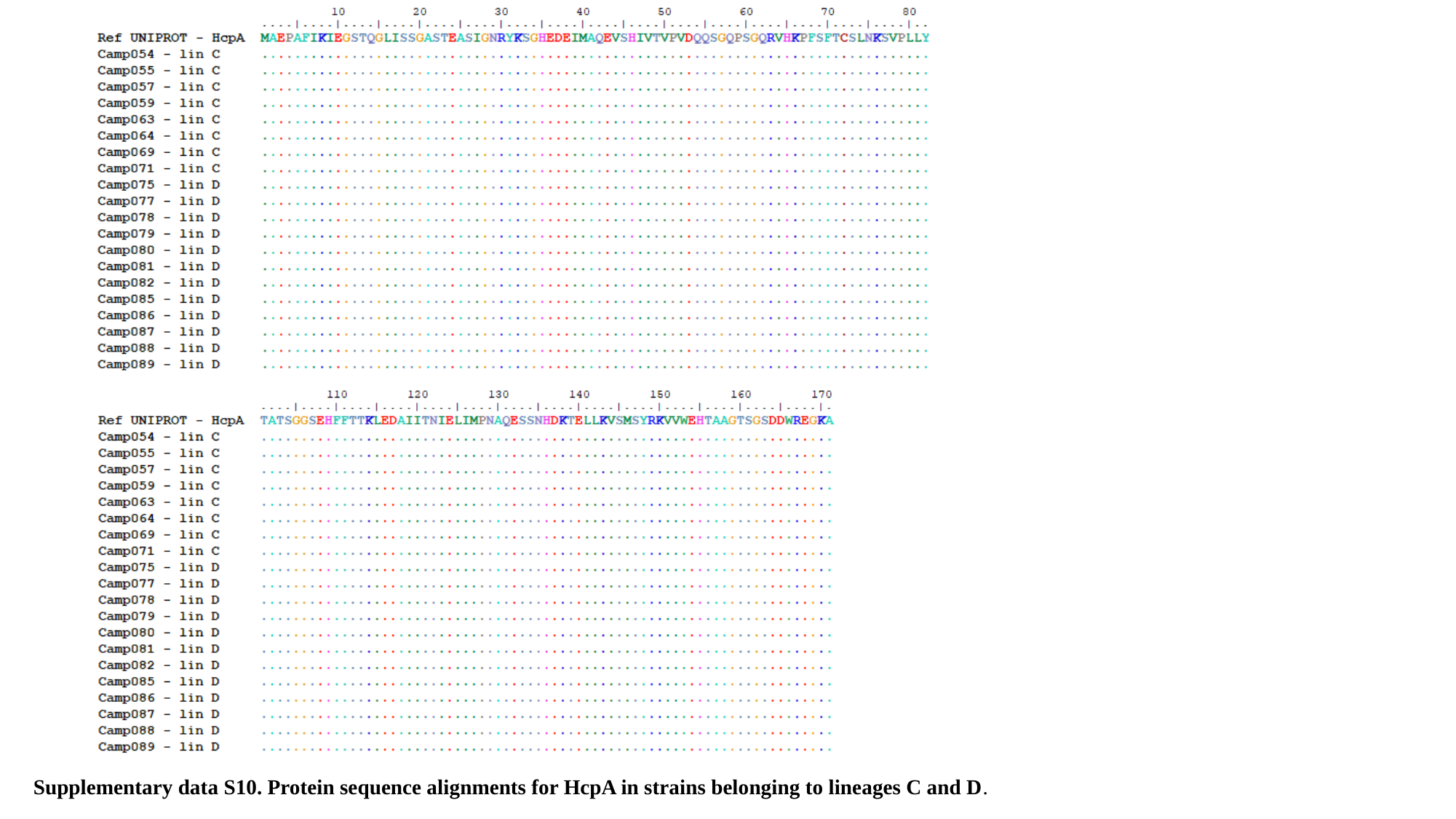

Supplementary data S10. Protein sequence alignments for HcpA in strains belonging to lineages C and D.
